# From stool to sequence: decoding the human diet with FoodSeq

**DOI:** 10.1128/msystems.00158-25

**Published:** 2025-06-23

**Authors:** Dorothy K. Superdock, Brianna L. Petrone, Michelle C. Kirtley, Lawrence A. David

**Affiliations:** 1 Department of Molecular Genetics and Microbiology, Duke University School of Medicine 12277 , Durham, North Carolina, USA; 2 Medical Scientist Training Program, Duke University School of Medicine 12277 , Durham, North Carolina, USA; Istanbul Medipol University School of Medicine, Istanbul, Turkey

**Keywords:** nutrition, genomics, DNA, sequencing, metabarcoding, diet, foodseq, biomarker, stool

## Abstract

Diet plays a pivotal role in human health and disease. Yet, nutrition studies have long relied on self-report methods for collecting dietary intake data despite known limitations. Although new technologies for dietary intake assessment and biomarker identification are in development, the integration of genomics has been limited. DNA metabarcoding, a method that identifies many taxa at once using a short region of DNA, has recently been adapted for use in stool samples from free-living humans. This process, called FoodSeq, provides an objective way to determine the foods people eat. FoodSeq has numerous advantages over self-report methods, is a necessary complement to other methodological innovations in dietary intake assessment, and holds considerable promise for application on an epidemiologic scale, enabling more robust analysis of global dietary patterns.

## DIET IS FUNDAMENTALLY TIED TO HEALTH

As one of the most complex and sustained environmental exposures of the human lifespan, diet has profound implications for health. Poor diet is the leading modifiable risk factor for mortality and morbidity in the United States (1) and responsible for 20% of deaths globally (2). The Global Burden of Disease study revealed that diet had surpassed cigarette smoking as the leading preventable risk factor for mortality worldwide (3). A growing body of evidence suggests specific dietary patterns can reduce the risk of certain diseases: the dietary approaches to stop hypertension (DASH) diet, which emphasizes fruits, vegetables, and low-fat dairy consumption, alongside reduced sodium intake, has been shown to lower blood pressure (4); high dietary fiber intake is associated with reduced risk of colon cancer (5); and a Mediterranean diet is linked to cardiovascular disease prevention (6). These are some of the most well-known examples of how diet can be a powerful tool for disease prevention and health management.

## SELF-REPORT METHODS OF DIETARY ASSESSMENT ARE LIMITED

Despite the importance of diet, challenges in obtaining accurate dietary intake data are well established. Self-reported dietary assessments presently provide the best methods feasible for tracking diet in most epidemiological studies, despite sources of error that were identified as early as the 1940s (7, 8). Common self-report methods widely used today include recalling foods consumed within the past 24 hours through prompts or interviews (24 hour recall), maintaining food diaries several days per week (food record), or completing questionnaires to summarize food consumption habits for up to a year (food frequency questionnaire [FFQ]). Though resulting data from these methods can be valuable, their use in nutritional epidemiology research has been intensely scrutinized (9, 10). Self-reports are subject to misreporting due to errors in human recollection (11), social desirability bias (12), imprecise portion size estimates (13, 14), insufficient capture of minority or ethnic foods (15), and the need for validation in each population of interest; thus, much effort has been invested in mitigating errors associated with self-reporting (16). These methods are further limited by their requirement for baseline levels of cognition or education (17), as well as time commitment and mental load, which contribute to overall participant burden.

A particularly challenging aspect of dietary tracking is the staggering number of potential food sources. The lists of recorded consumed plant species alone range from the hundreds (354 of commercial importance; 866 crops [18]) to the thousands (4,079 compiled from published lists [19]) out of tens of thousands of theoretically edible wild plants. Over 2,000 species of fungi are reported to be edible (20), as are 13,000 species of fish raised in fisheries (21), and dozens of livestock species and animal-derived products. The U.S. Department of Agriculture’s FoodData Central, which is the main source of food composition data in the United States, contains over 400,000 food items as of September 2024 (22). These complexities multiply when aggregating data across hundreds of global nations, languages, and cultures. How is it possible to collect consistent data on such a complex exposure?

## CALLS FOR TECHNICAL INNOVATION TO IMPROVE DIETARY ASSESSMENT

Calls for innovation in dietary assessment have prompted new tools and techniques for collecting dietary data, beyond self-reports. Many fall into two broad categories: (i) improving the accuracy of traditional dietary data and (ii) identifying biomarkers of food intake.

Efforts to improve accuracy, reduce biases, and streamline data collection in dietary assessment have leveraged various technological and methodological advancements. These methods include but are not limited to the following innovations: web-based 24 hour recalls, such as the widely used ASA24 (23); digital photography methods, including AI-powered image analysis (24, 25); and wearable sensor devices, personal digital assistants, and barcode scanning technologies (26, 27).

Researchers have also developed objective biomarkers of food intake to complement or validate self-reported data. The doubly labeled water method is considered the gold standard for measuring total energy expenditure (28, 29). Urinary nitrogen has been established as a reliable marker for protein intake (30), skin carotenoid levels a proxy for fruit and vegetable consumption (31, 32), and carbon and nitrogen stable isotope ratios in hair and blood have been used to indicate consumption of animal protein (33, 34) and sweeteners (35).

We present strengths, limitations, and distinguishing features of several approaches in Table 1.

**TABLE 1:** Strengths and weaknesses of select approaches for dietary assessment

Feature	FFQs, records, recalls (36)	Image-based methods (24, 25)	Metabolomics (37, 38)	MEDI (39)	FoodSeq (40–43)
Avoids memory bias	No	Yes	Yes	Yes	Yes
Avoids literacy and cognitive requirements	No	Sometimes	Yes	Yes	Yes
Adherence to method	Challenging	Easy	Moderate	Moderate	Moderate
Data harmonization	Challenging	Variable	Variable	Easy	Easy
Enumerates nutrients	Yes	Yes	Yes	Yes	Potentially
Enumerates foods	Yes	Yes	Sometimes	Yes	Yes
Detects unseen components	Sometimes	No	Yes	Yes	Yes
Detects low-abundance components	Yes	Uncertain	Sometimes	Sometimes	Yes
Detects food processing	Yes	Variable	Sometimes	Sometimes	Sometimes
Can collect retrospective data	Sometimes	No	Yes	Yes	Yes
Low cost	Sometimes	Sometimes	No	Sometimes	Yes
Validation	Yes	Moderate	Moderate	Moderate	Moderate
Quantitative	Yes	Yes	Sometimes	Moderate	Moderate
Non-invasive sampling	Yes	Yes	Variable	Yes	Yes
Scalable	Variable (FFQ only)	Yes	Yes	Yes	Yes

There has been a particular interest in exploring metabolomics methods for identifying novel biomarkers of dietary intake (37, 38, 44). Numerous new insights have emerged from nutritional metabolomics, such as correlations between metabolites and foods (45); metabolite biomarkers for dietary patterns associated with reduced risk of chronic diseases, like cardiovascular disease and type 2 diabetes (46–48); and associations between metabolites and diet quality (49, 50). Despite these advances, much of the chemical complexity of the diet remains unmapped (51): fewer than 2% of mass spectra are typically annotated using existing databases (52), and most downstream analyses focus only on known metabolites. Even when known, metabolites can be variably named, causing difficulty in review and synthesis of findings (53), and non-specific, for example, carotenoids and vitamin C are common to numerous fruits and vegetables and consequently are impossible to map to individual foods (54). Thus, efforts to improve food annotations are ongoing (55, 56). Additionally, only a limited number of studies have demonstrated the use of dietary metabolomics as a quantitative tool (57). We place metabolomics within the context of other dietary assessment methods in Table 1. Given these gaps, there remains substantial, unexplored potential for advancing dietary data through the integration of techniques that *directly* analyze whole food consumption.

## FOOD DNA IS A DIETARY BIOMARKER

The need for alternative approaches to measure diet has long been apparent to animal researchers, and biomarkers of diet have been used in these settings for decades (58). Zoologists and ecologists have never been able to ask their subjects what they ate and previously had to rely on cumbersome observational field studies of animal eating behavior and microscopy of animal feces to identify partially digested food species by their morphology (59). With the advent of sequencing technologies and early microbial work establishing gene sequence divergence for species identification (60, 61), researchers across disciplines began PCR-amplifying short “barcode” segments from samples—using primers matching conserved regions of DNA flanking variable segments—and then sequencing mixed barcode pools (“metabarcoding”) to identify the organisms present (62–65). Metabarcoding methods were first adapted to the study of diet in animal species whose diets were difficult or impossible to examine with existing techniques (59). Use of dietary DNA metabarcoding has since expanded to include studies of the diets of >250 wild species (66, 67), including mammalian dietary generalists like wild pigs (68) and brown bears (69).

Outside of its application to fecal samples to investigate animal diets, DNA metabarcoding is used for many applications. DNA metabarcoding has been used to assess the prevalence of mislabeled meat and poultry products (70), identify aquatic species in commercially available imitation crab products (71), detect seafood fraud (72), map the origin of honey by its floral composition (73, 74), and predict a wine’s vineyard of origin (75). The U.S. Food and Drug Administration uses DNA metabarcoding for fish regulation (76), and commercial services offer detection of plants and animals in complex foods (77). These techniques have even been employed in human samples, where microbiome research has made commonplace a type of DNA metabarcoding: 16S rRNA gene sequencing.

## FoodSeq: DNA METABARCODING FOR HUMAN DIETARY ASSESSMENT

Although human diets are derived from the tissues of other living organisms in the plant, animal, and fungal kingdoms, the use of DNA as a biomarker for human dietary intake has remained largely overlooked as a tool in living human populations, despite its ability to complement limitations in current methods and its utility and widespread application in other fields. While it may seem a short conceptual step to apply metabarcoding strategies developed for animals and foods to obtain dietary data from living humans, DNA metabarcoding methods have primarily been applied to the analysis of stomach contents during autopsy (78), calcified plaque from teeth collected from roughly 100- to 200-year-old burial sites (79), and intestinal contents of a >5,000-year-old Neolithic mummy (80). In other words, in parallel to wild animal studies, DNA metabarcoding methods have been limited to human contexts that lacked alternative ways to collect diet data.

This limited use of DNA metabarcoding in human nutrition may stem from the history and evolution of nutrition science. Once a field rooted in biochemistry, nutrition scientists naturally pursued metabolomics for the exploration of dietary metabolites (81). Now, modern nutrition science has evolved to incorporate a broader range of disciplines. In particular, the growing interest in the relationships between diet, gut microbes, and metabolism (82–86) over the last two decades has led to a convergence of ideas: can we study food DNA in human stool the same way we study microbial DNA?

**Fig 1:**
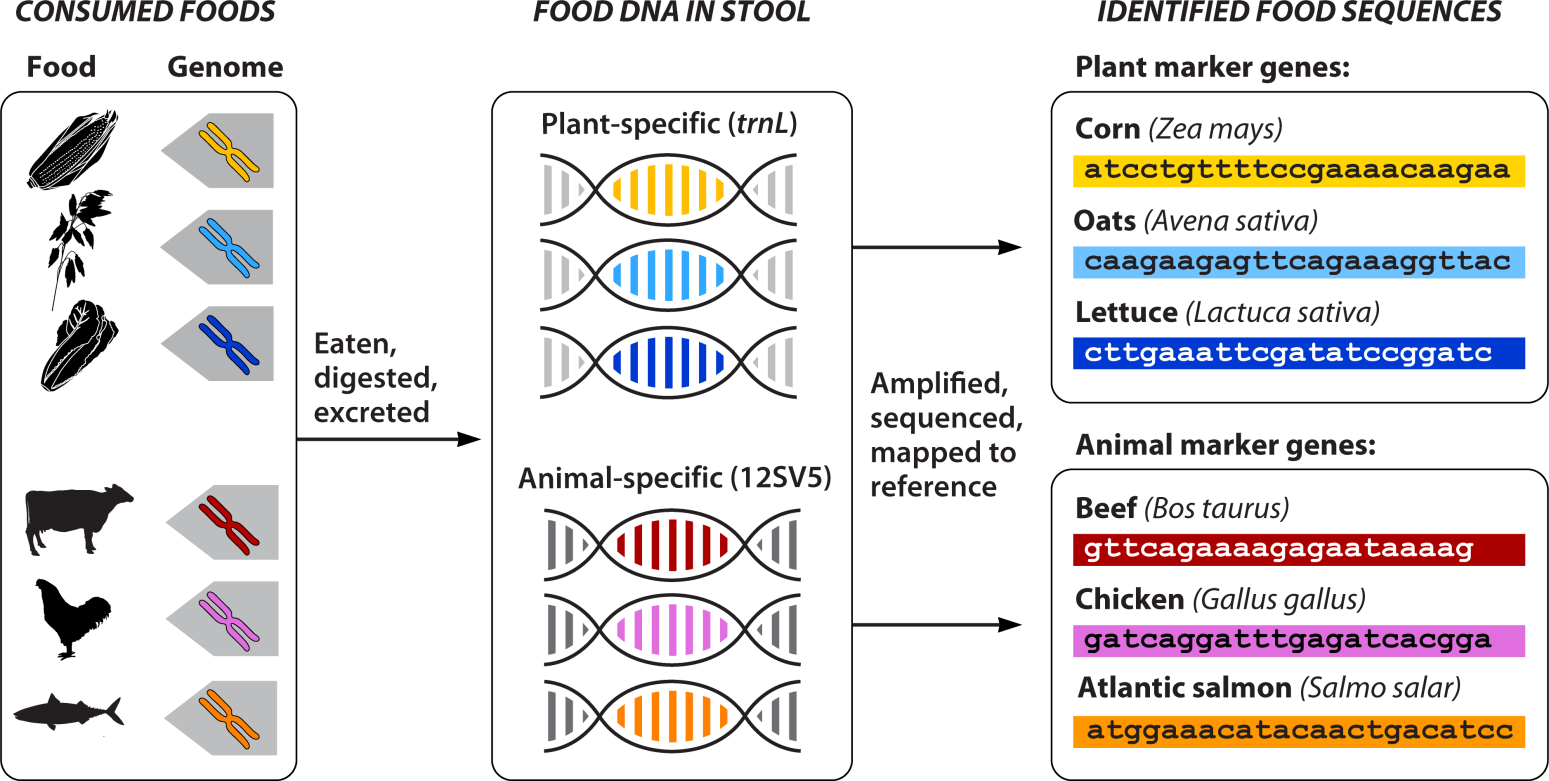
Overview of FoodSeq workflow. Conserved primers flanking variable regions of plants (P6 loop, g/h region, of the chloroplast *trnL* gene) and animals (V5 region of the 12S mitochondrial rRNA gene), amplify dietary DNA in human fecal samples to reconstruct dietary intake.

Leveraging our expertise in microbiome research, our group has pioneered the use of DNA metabarcoding for dietary intake of plants and animals in modern, free-living people (Fig. 1). We have demonstrated that DNA metabarcoding is not just a last resort for impossible dietary assessments but a viable and valuable tool even when traditional dietary surveys are possible (40–42). We use primers that target the chloroplast *trnL* intron (40, 41) and mitochondrial 12S rRNA gene (42), mapping dietary plant and animal DNA sequences to a custom database of reference sequences that we have curated (Fig. 1). We have given the name FoodSeq to the process of dietary DNA metabarcoding in human stool for several reasons. First, the name makes the concept easy to understand: we are sequencing food DNA in stool. Second, researchers in many fields are not familiar with the term “DNA metabarcoding.” We sought to create a simple term that could be used across various disciplines.

## FoodSeq HAS KEY CONCEPTUAL STRENGTHS

- Unlike most dietary survey methods, FoodSeq can overcome differences in age, cognition, socioeconomic status, literacy, language, and culture. Global comparisons across studies that were previously difficult, if not impossible, due to differences in self-report methods (36), are now feasible. FoodSeq enables access to data from cohorts including infants, young children, and older adults—populations that currently have limited diet data (87) due to challenges with completing surveys.
- FoodSeq has the potential to identify more foods than traditional dietary surveys. Since FoodSeq does not rely on prior knowledge of which foods are likely to be consumed in different cultures, the risk of overlooking regionally or culturally specific items that could be missed by broad surveys is minimal. FoodSeq can also detect trace ingredients that may be missed using current methods, such as herbs and spices.
- We can perform non-invasive, retrospective assessment of dietary intake wherever stool was previously biobanked. We use the same stool storage and DNA extraction methods commonly used for gut microbiome research; therefore, any samples from these studies can theoretically be used for dietary intake analysis via FoodSeq. Well-preserved DNA can last thousands of years (80) and is not sensitive to decay in the same way metabolites can be. Retrospective experiments using existing fecal samples or DNA could synchronize dietary and microbiome data to uncover new diet-microbiome relationships (88), and future studies can be designed prospectively to query microbiome and diet in parallel.
- Computational and statistical methods devised for microbiome analysis can now be adapted for nutrition analysis. Data generated from FoodSeq and 16S rRNA gene sequencing are similar in nature; thus, established bioinformatic and statistical tools from the microbiome field enable us to analyze patterns in the human diet. The rapid growth of microbiome research over the past decade has led to the development of robust, open-source computational pipelines and statistical frameworks (89, 90). By leveraging these existing tools and methodologies, FoodSeq can benefit from years of computational advancements in a related field, potentially accelerating its analytical capabilities and adoption by researchers already familiar with microbiome data analysis.
- FoodSeq is economical and scalable. For researchers experienced with genomic sequencing, direct in-lab expenses for an integrated FoodSeq and microbiome workflow can be below $100 per sample, from DNA extraction through sequencing. In contrast, the cost of untargeted metabolomics typically starts at $100 per sample at a minimum and is commonly two to three times higher for samples submitted by external users (91–93). As the cost of genomic sequencing continues to trend downward (94), FoodSeq’s scalability will only grow with time. Additionally, FoodSeq allows the field of nutrition to share in the benefits of technological advances in genomics. As sequencing becomes more cost-effective, FoodSeq could also be used to routinely monitor dietary intake, especially in clinical settings where stool samples may already be collected.
- Finally, FoodSeq yields more intuitive results because it identifies food species rather than unseen nutrients. While some nutrients are well understood by the public, many remain abstract concepts, leading public health institutions to translate nutrient-based recommendations into food-based guidance, such as the Dietary Guidelines for Americans (95) and MyPlate (96). FoodSeq aligns naturally with this food-centric approach by directly identifying food species in biological samples, generating results that are inherently more relatable to how people think about their diets (97). This food-species-forward presentation of dietary data has the potential to make research findings more readily accessible to the public, while still allowing nutrients to be inferred from the identified species and patterns.

We note the distinction between our use of amplicon-based DNA sequencing to track food intake and metagenomic sequencing performed directly on food with heterogeneous taxon composition (e.g., sausage meat), a technique called “All-Food-Seq” (98), which is designed for monitoring food quality rather than dietary intake. We also note that our use of genomics is distinct from how the term is used in nutrition science. Nutritional genomics, or “nutrigenomics,” has focused on the interactions between nutrients and human genes, including both the effects of nutrients on gene expression (99) and the effects of genetics on nutrient metabolism (100). However, here, we use genomics to identify the genes of consumed foods.

Shotgun metagenomics has also been used in the food and nutrition space. Researchers have used metagenomic sequencing of food samples to authenticate food ingredients and identify contaminants (98, 101). Another group has employed the indirect approach of using metagenomic data to develop microbial metagenomic profiles as biomarkers of food intake (102). Finally, in the most similar vein to FoodSeq, another group has developed a pipeline to analyze dietary intake by annotating individual food-derived reads from stool metagenomic data (39). This approach, known as “metagenomic estimation of dietary intake,” or MEDI, has several strengths: it may be less subject to amplification biases than amplicon-based methods (103), and it can be applied to already existing shotgun datasets (39). However, the depth of sequencing required to identify low abundance food genomes (30 million reads per sample)—present at much smaller amounts than bacterial and human DNA—could be cost-prohibitive for many research groups. Based on current estimates, MEDI costs approximately $300 per sample. Still, MEDI could be applied to sequences from previously deeply sequenced fecal metagenomic datasets, many of which are publicly available in databases like the National Center for Biotechnology Information Sequence Read Archive. These metagenomes already include microbial sequences, providing additional value. Separately, it is worth noting that MEDI also demands substantial computational resources: its reference database is large (500 GB), and without appropriate decoy genomes, there is a risk of misclassifying host or microbial reads as food sequences. In contrast, our amplicon-based approach benefits from strictly curated, food-specific reference databases and a less computationally intensive bioinformatic workflow. We compare MEDI to FoodSeq and other dietary assessment methods in Table 1.

## MILESTONES ACHIEVED THROUGH FoodSeq TO DATE

**Fig 2:**
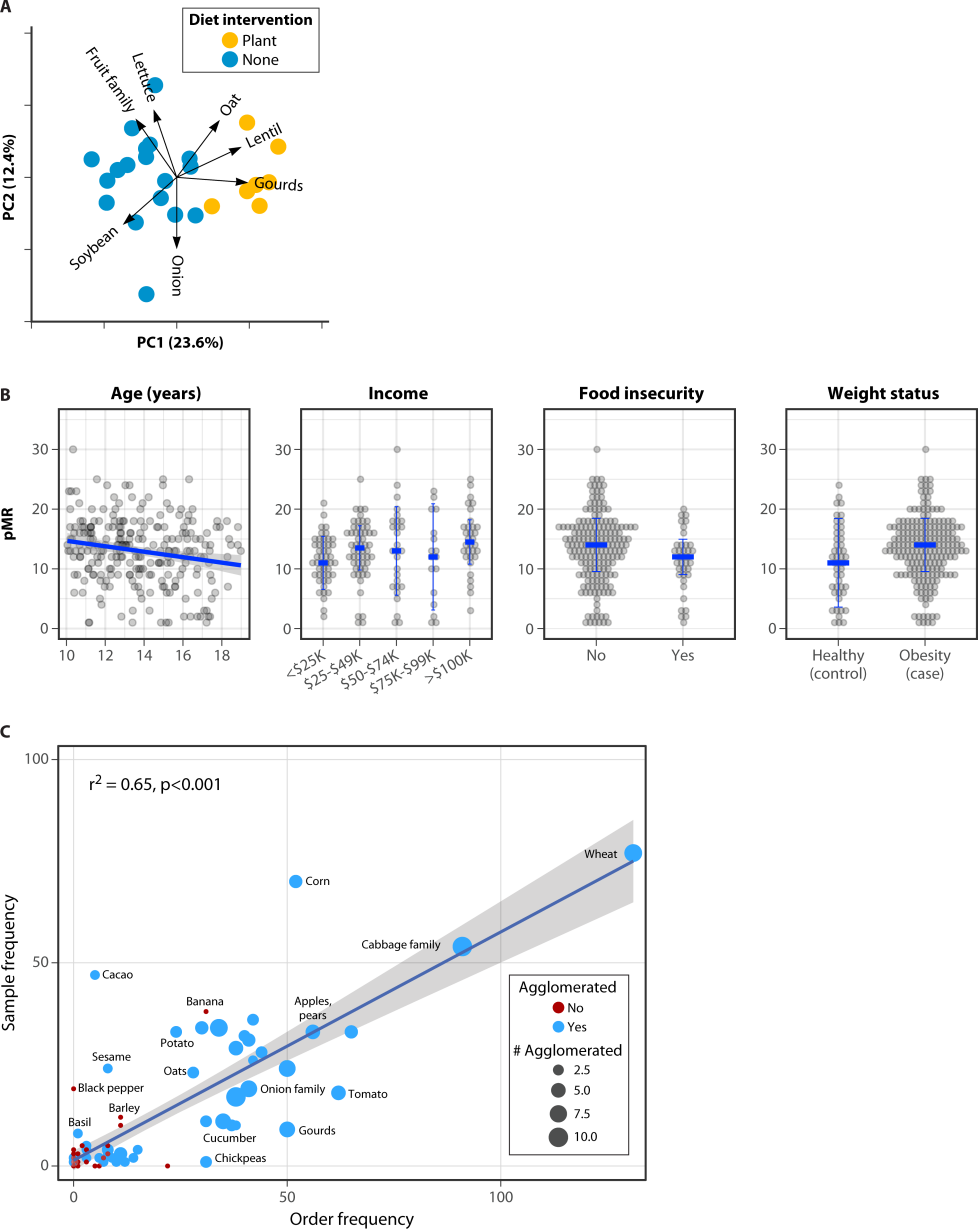
Selected FoodSeq highlights: study arm clustering, richness associations, and taxon-food concordance. (**A**) Principal component analysis of dietary plant taxa composition in participants from experimental and non-interventional study arms. Each point represents an individual fecal sample, colored by sample collection timepoint (yellow: sample collected during plant-based diet intervention; blue: sample collected during a non-intervention/free-eating timepoint). The biplot indicates the top seven plant taxa contributing most to the separation along principal components PC1 (23.6%) and PC2 (12.4%). (**B**) The number of unique plant taxa identified by FoodSeq (pMR) captures known relationships between dietary diversity and demographic, health, and socioeconomic variables. Raw data underlying significant or trending covariates from a multiple regression where adolescent pMR was negatively associated with age (β= −3.9 [95% CI −7.1 to −0.7], *P* = 0.02), positively associated with higher income categories (β = 2.2 [0.03 to 4.4], *P* = 0.05 for $25,000 to 49,000/year and β = 3.0 [0.1 to 5.9], *P* = 0.04 for $100,000/year, both relative to lowest bracket of <$25,000/year), and trended lower with food insecurity (β = −1.7 [−3.7 to 0.3], *P* = 0.09) and higher with obesity status (β = 1.8 [−0.1 to 3.7], *P* = 0.06). (**C**) High concordance between plant taxa identified by FoodSeq and corresponding grocery foods. Linear regression (adjusted *r*^2^ = 0.65, *P* < 0.001). Foods mapping to multiple FoodSeq taxa were counted for each applicable taxon and taxa mapping to multiple foods counted for each applicable food (“agglomerated,” blue dots, number of items agglomerated indicated by size of the dot). Plant taxa with only one distinct corresponding food item are represented by red dots.

In our proof-of-concept work, we provided the first evidence that DNA metabarcoding could be adapted as a tool for human dietary pattern assessment (41). Here, we applied FoodSeq using *trnL* primers to a small set of stool samples that had been biobanked as part of a previous dietary intervention study. We were able to distinguish when participants were in experimental or non-interventional diet arms of the study based on their FoodSeq data (Fig. 2A). For individuals consuming a controlled, plant-rich diet, we saw a 70% PCR success rate. And, we showed it was feasible to detect consumed plant species that were present in diet records kept by participants (41).

In follow-up work, we optimized our protocol, resulting in a PCR success rate of 92% (40), which exceeds response rates for validated FFQs (76%–82%) (104). In stool samples from a weight-loss dietary intervention study with available cafeteria records, we were able to detect a strong concordance between plant DNA and menu data, even though participants were also known to be consuming off-menu foods. For each sample, the number of unique plant taxa detected in stool using FoodSeq was positively correlated with the average number of plants in menus from the 2 days prior to stool collection (Spearman ρ = 0.42, *P* = 0.03) (40). We also showed that FoodSeq can complement weak or non-existent dietary survey data. In a cohort of adolescents who were unable to fill out dietary assessment measures, we were able to identify 111 unique *trnL* sequence variants originating from 46 plant families, 85 plant genera, and 72 plant species (40).

FoodSeq also shows promise as an epidemiological tool. Just as richness is a useful diversity metric in microbial ecology, we found that metabarcoding-derived, molecular dietary diversity metrics (i.e., plant metabarcoding richness, pMR, the number of unique plant species observed) can be valuable for testing epidemiological hypotheses. We were able to test the relationships between dietary diversity and several demographic, biological, and socioeconomic variables (40). In an adult cohort, we found that pMR was positively correlated with the plant-based component of the Healthy Eating Index 2015 and the healthful component of the plant-based dietary index (hPDI), both standard measures of diet quality (40). In an adolescent cohort, pMR was found to be negatively associated with age, consistent with reports that adolescents are more likely to be eating apart from family as they age (105) (Fig. 2B). We observed that adolescent pMR was both positively associated with higher income categories and trended lower with food insecurity (40), in line with epidemiologic findings of increased dietary diversity with higher socioeconomic status (106, 107) (Fig. 2B). We also found that pMR in the adolescent cohort also tended to be higher with obesity, a finding that agrees with reports that greater dietary diversity is not always better and could be due to higher consumption of ultra-processed foods, refined grains, and sugar-sweetened beverages instead of healthful dietary patterns (108, 109) (Fig. 2B). Of note, we would not have been able to test these associations in the adolescent cohort without FoodSeq, as comprehensive dietary questionnaires were too onerous for families to complete in this context.

Additionally, we showed that just two to three samples per person are enough to detect interindividual variation (40). In two adult longitudinal cohorts, significant correlations persisted between plant metabarcoding richness and two validated dietary indices in a majority of subsampling trials, when reducing to three samples per individual in one cohort and two samples per individual in the other. These results indicate that even with a limited number of samples, FoodSeq can be used to differentiate individuals similarly to the ranking of individuals by a traditional dietary diversity index (food variety score [FVS]) and a dietary quality index (hPDI) (40). Accordingly, we recommend collecting at least two to three samples when possible. Collecting several stool samples for FoodSeq thus resembles the usual practice of measuring habitual diet over several days when using 24 hour diet recalls or diet records to account for intraindividual day-to-day variations in food consumption (110) (e.g., NHANES collects 2 days of dietary data intake 3–10 days apart [111]).

FoodSeq has also shown value in prospective, randomized controlled trials. In Aqeel et al. (43), we assessed the impact of a grocery store intervention vs usual care for improving diet quality in children with obesity and found the frequency of plant species detection using FoodSeq strongly correlated with the specific food items purchased in grocery orders (adjusted *R*^2^ = 0.65, *P* < 0.001) (Fig. 2C) (43). This highlights FoodSeq’s effectiveness as a tool to assess adherence to dietary interventions. Furthermore, because FoodSeq data are in the form of DNA sequences, we were able to easily combine the data from this study with data from a larger, closely related cohort (112). Using this larger sample set as our reference comparator, we were able to detect dietary shifts in the grocery intervention group, but not the usual care group, toward nutrient-dense fruit and vegetable taxa during the treatment period. We note that the standardized nature of DNA sequence data enabled this cross-study comparison, which would have been challenging or impossible with traditional dietary assessment methods due to differences in data collection and formatting across studies.

Finally, we have shown that FoodSeq can indeed be successfully paired with another omics method to improve dietary data. In Petrone et al. (42), we applied FoodSeq to identify dietary DNA along with metaproteomics to identify dietary proteins reflective of both dietary plant and animal consumption. We found significant overlap in food taxa identified by FoodSeq, metaproteomics, and diet records, providing additional validation of food items consumed (42). When comparing FoodSeq to metaproteomics in detecting items from diet records from 2 days prior, we found that FoodSeq could better predict individual food taxa. However, metaproteomic data integrated with FoodSeq data provided more dietary information than DNA alone. Through metaproteomics, we were able to detect tissue-specific dietary proteins like myosin, ovalbumin, and beta-lactoglobulin, suggesting the possibility of differentiating between food types derived from the same animal, such as beef vs milk or chicken vs egg (42). In some cases, proteomics resulted in higher taxonomic resolution for specific items such as differentiating durum wheat from bread wheat. Altogether, these data show promise in a combined omics approach to enhance the resolution of biomarker-based dietary assessment.

## PROSPECTUS

### The ongoing evolution of FoodSeq

The complexity of human dietary patterns presents unique challenges for FoodSeq. Individual tastes and attitudes, cultural and societal influences, and socioeconomic status contribute to diverse eating habits (113, 114), making human diets far more varied than those of other animals. Our species’ unique way of cultivating, storing, preparing, and distributing foods, coupled with rapid changes in global food systems (19, 115, 116), adds further intricacies. In light of the complexity of the human diet, FoodSeq is substantially more challenging than DNA metabarcoding for dietary analysis in animal studies. Recognizing these considerations, we identify several areas for further technical advancement and validation.

While our previous studies have demonstrated the efficacy of FoodSeq for measuring overall dietary diversity (40) and detecting specific foods (41, 42), further validation would be beneficial. In particular, FoodSeq’s performance should be evaluated across a broad spectrum of individual foods, considering various preparation methods. Our data support previous literature that suggests DNA, particularly smaller fragments, can survive various cooking temperatures (117–119). We consistently detect DNA from foods that are usually cooked, such as wheat, rice, chicken, and pork. Encouragingly, we have also successfully detected DNA from guar beans, the source of guar gum, a widely used thickening agent in highly processed foods (120), and tea, indicating that processes like grinding and brewing do not eliminate all detectable DNA. Similarly, MEDI has detected DNA postulated to be from plant-derived bulking agents and food dyes, which are common additives in extensively processed food products (39, 121). While these initial findings suggest DNA survives cooking and processing, systematic studies are required to fully understand the impact of various food processing techniques on DNA detectability and to establish the range of FoodSeq, particularly among highly processed foods.

Another area for further development is quantitation. Literature already exists in the ecological context on the potential to treat sequencing reads quantitatively (122), and these approaches should be adapted to human dietary analysis. Data from testing (123) or feeding (124–126) controlled mixtures have suggested that quantitation performance depends on (i) the consuming species, (ii) the consumed species, and (iii) the complexity and size of the mixture. Further quantitation work to enable estimates of the absolute amount of food DNA in stool could incorporate “spike-ins” of known quantities of an internal standard before DNA extraction (127) or copy number standards during PCR. Additionally, developing calibration methods to account for amplification biases (103, 128) will be crucial for improving the quantitative accuracy of FoodSeq.

Current limitations in taxonomic resolution also warrant attention. FoodSeq sometimes characterizes closely related food taxa only at higher levels. For example, within the species *Brassica oleracea*, the closely related cultivars broccoli, cauliflower, and Brussels sprouts are not yet differentiated using our *trnL* primers. While this broader classification may not meaningfully impact some dietary studies (e.g., cruciferous vegetables are often evaluated as a group), improving resolution could provide valuable insights in certain research contexts. Similarly, the 12SV5 primers we currently use for animal residues do not capture invertebrate species, such as shellfish. In both cases, developing protocols with primers targeting alternative gene regions could address these limitations, potentially expanding FoodSeq’s applicability to a wider range of research questions.

### The revolutionary potential of omics-driven diet tracking methods

New omics methods for dietary tracking will transform the way we study diet. We expect the identification of new, species-based dietary patterns will reveal previously unrecognized links with health outcomes. Already, these methods are uncovering insights about the role of diet in contexts like inflammatory bowel disease (129), immune response to SARS-CoV-2 vaccination (121), and infant microbiome maturation (88). By providing unprecedented access to dietary data wherever fecal samples have already been collected or are accessible in the future, genomics methods offer distinct advantages in capturing the complexity of human diets across diverse populations and are an integral complement to other omics methodologies that are already more widespread in nutrition analysis. In clinical settings, routine monitoring of patients’ fecal samples alongside relevant outcome measures could enable evidence-based dietary adjustments to improve recovery. In nutrition intervention studies, genomics methods could be used to evaluate adherence to a specified dietary protocol. On a global scale, genomic dietary data have the potential to transform nutritional epidemiology. Looking ahead, we expect these genomic insights will be instrumental in advancing our understanding of diet-related health outcomes and shaping future dietary recommendations to enhance nutritional adequacy, food security, and sustainability.
